# Transcriptome analysis of goat adipose tissue-derived mesenchymal stem cells cultured in variable oxygen conditions

**DOI:** 10.3389/fcell.2026.1814793

**Published:** 2026-07-01

**Authors:** Utkarsha Vishwakarma, Michelle Abraham, Ibraz Kori, Aakash Chawade, Sandeep Kumar Kushwaha, Sandeep Goel

**Affiliations:** 1 Biotechnology Research and Innovation Council (BRIC)-National Institute of Animal Biotechnology (NIAB), Hyderabad, Telangana, India; 2 Regional Centre for Biotechnology (RCB), Faridabad, Haryana, India; 3 Sveriges Lantbruksuniversitet, Alnarp, Sweden

**Keywords:** adipose-derived mesenchymal stem cells, ADSC-based therapy, hypoxia, RNA sequencing, transcriptome analysis

## Abstract

**Introduction:**

Oxygen tension influences mesenchymal stem cell biology, but the transcriptional responses of goat adipose tissue-derived mesenchymal stem cells (gADSCs) to different oxygen-exposure conditions remain incompletely understood.

**Methods:**

RNA sequencing (RNA-seq) was used to investigate oxygen-dependent transcriptional responses in gADSCs cultured under normoxia (NO), sustained hypoxia (HO), and transient hypoxia (THO). Differentially expressed genes (DEGs), enriched biological processes, and protein-protein interaction networks were analysed. Selected DEGs and hub/bottleneck genes were validated by quantitative reverse transcription PCR (RT-qPCR).

**Results:**

The analysis identified condition-associated gene expression changes and candidate pathways related to cell-cycle regulation, DNA repair, extracellular matrix organisation, inflammatory response, pH regulation, angiogenic signalling, and hypoxia-inducible factor-associated adaptation. RT-qPCR validation further revealed differential regulation of hypoxia-inducible factor 1-alpha (HIF1A) and hypoxia-inducible factor 2-alpha (HIF2A), suggesting possible divergence between acute and sustained hypoxic responses.

**Discussion:**

These findings provide a transcriptomic resource for understanding oxygen-dependent regulation of gADSCs. However, protein-level validation and functional assays are required to confirm the biological roles of the prioritised genes and assess their relevance to regenerative applications.

## 1 Introduction

Mesenchymal stem cells (MSCs), especially adipose tissue-derived mesenchymal stem cells (ADSCs), have garnered attention in regenerative medicine due to their multipotency, immunomodulatory properties, and ease of isolation (Finocchio et al., 2023). These cells are promising for clinical applications, including tissue repair, modulation of inflammation, and immunotherapy (Wang et al., 2011). A key factor influencing ADSC behaviour and function is the oxygen microenvironment in which they reside (Samal et al., 2021). Oxygen tension plays a central role in cellular metabolism and regulates stem cell fate decisions, such as proliferation, differentiation, and self-renewal (Mas-Bargues et al., 2019).

Under physiological conditions, MSCs experience oxygen levels ranging from 1% to 7%, significantly lower than atmospheric oxygen concentrations (21%) (Di Mattia et al., 2021). This hypoxic environment, common in physiological niches such as bone marrow and adipose tissue, profoundly impacts MSC behaviour by enhancing stemness and regenerative potential (Mahjoor et al., 2023). Exposure to hypoxia has been shown to promote the secretion of trophic factors, cytokines, and extracellular vesicles, contributing to tissue repair and regeneration (Yang et al., 2022). In contrast, transient fluctuations in oxygen levels, such as those during ischemia or tissue injury, can also alter MSC behaviour. MSCs respond to transient hypoxia by activating adaptive mechanisms to cope with oxygen deprivation, modulating their gene expression profiles and phenotypic characteristics (Jiang and Scharffetter-Kochanek, 2020). Understanding how MSCs adapt to dynamic oxygen conditions is crucial for optimising their therapeutic potential.

Transcriptomic analysis is a valuable tool for elucidating the molecular mechanisms underlying MSC responses to different culture conditions (Cruz-Barrera et al., 2020). Although numerous studies have examined MSCs under hypoxia to assess angiogenic, anti-inflammatory, immunomodulatory, and regenerative properties (Ganguly et al., 2023; Elabd et al., 2018), most have focused on short-term hypoxic exposure. Data on prolonged hypoxic priming, particularly in ADSCs, remain limited. Whole-transcriptome analysis can identify genes, regulatory networks, signalling pathways, and molecular targets that mediate MSC adaptation to normoxic, hypoxic, and transient oxygen conditions.

In this study, we used a whole-transcriptome-based approach to examine the effects of variable oxygen conditions on gADSCs. Specifically, we compared normoxia (NO; 72 h at 21% O_2_), transient hypoxia (THO; 72 h at 2% O_2_), and sustained hypoxia (HO; three passages under 2% O_2_). We analysed gene expression profiles to identify molecular mechanisms associated with gADSC adaptation to different oxygen environments. These findings contribute to a better understanding of MSC biology and may inform future strategies to optimise MSC-based therapies in regenerative medicine and tissue engineering.

## 2 Materials and methods

Unless stated otherwise, all reagents and chemicals were procured from Sigma–Aldrich (Bangalore, Karnataka, India).

### 2.1 Primary isolation and culture of gADSCs

The isolation and culture of goat adipose-derived mesenchymal stem cells (gADSCs) were performed as previously described (Abraham et al., 2024; Abraham and Goel, 2024). In brief, adipose tissue (AT) was aseptically collected from the inguinal region of freshly slaughtered goats (*Capra hircus*; n = 3) at a local slaughterhouse. The excised tissue was immediately transferred to phosphate-buffered saline (PBS) supplemented with 1× antibiotic-antimycotic solution (AA, 100 U/mL penicillin, 0.1 mg/mL streptomycin, and 0.25 μg/mL amphotericin B) and transported to the laboratory on ice under sterile conditions. After arriving at the laboratory, the AT was rinsed with PBS and kept in PBS containing AA with mild shaking at 4 °C to eliminate contamination. After 1 h, the AT was transferred to the biosafety cabinet and washed 3 times with PBS. Approximately 5 g of AT was placed in a sterile Petri dish and minced thoroughly into small pieces (~1 mm^3^) using sterile scissors. Minced tissue was transferred into a 50 mL centrifuge tube and resuspended in a digestion mixture containing 1 mg/mL collagenase type II (Gibco) in PBS. The digestion mixture was incubated at 37 °C in the shaking water bath (100 rpm) for 30 min. After the tissue digestion, the digestion mixture was filtered through a 100 μm cell strainer (Corning). The filtrate was centrifuged at 726 *g* for 5 min, yielding a stromal vascular fraction (SVF) cell pellet. The supernatant was discarded, and the SVF cells were resuspended in 5 mL PBS supplemented with 10% fetal bovine serum (FBS; Gibco) and AA. The cell pellet was spin-washed 3 times with PBS and resuspended in erythrocyte lysis buffer for 2 min. The reaction was halted by adding twice the volume of PBS with 10% FBS. The cell count was performed using a haematocytometer (improved Neubauer counting chamber, Germany), and cell viability was assessed by the trypan blue dye exclusion method. The cells were seeded at a density of 2 × 10^4^ cells/cm^2^ in Dulbecco’s Modified Eagle Medium/Ham’s Nutrient Mixture F12 (DMEM/F12) supplemented with 10% FBS (Gibco) and 1× AA (complete medium) at 37 °C in a humidified 5% CO2 environment. The medium was replenished every 48 h. Once the confluency of seeded cells reached approximately 80%–90%, cells were trypsinised and subcultured. Briefly, the confluent cells were washed twice with PBS and treated with a working trypsin-EDTA solution (0.05% trypsin and 0.02% EDTA) for 2–5 min in a humidified environment at 37 °C, and trypsin activity was halted with a complete medium (DMEM/F12 containing 10% FBS with 1×AA). The recovered cells were centrifuged at 726 *g* for 5 min and subcultured. The cells in passages 3-5 were characterised to confirm their identity as MSCs. All subsequent experiments were conducted in passages 6-9.

### 2.2 MSC-specific surface markers analysis of the gADSCs

Flow cytometry was used to assess the immunophenotype of gADSCs. Cells were harvested by trypsinisation, as described earlier (Abraham et al., 2024). Briefly, for each antibody, 1 × 10^6^ gADSCs were fixed in ice-cold ethanol for 30 min on ice, washed three times with PBS, and suspended in blocking buffer (0.5% BSA with 2% FBS in PBS). Cells were incubated overnight at 4 °C with FITC-conjugated primary antibodies against CD90, CD73, CD105, CD31, CD45, and CD34 (BioLegend) at the manufacturer-recommended dilutions. Cells stained with FITC-conjugated isotype-control IgG were used as negative controls. The labelled cells were washed three times with FACS buffer (0.5% BSA and 0.05% sodium azide in PBS), resuspended in 500 µL FACS buffer, and analysed on a BD FACS Melody using FlowJo 10.8.1 software.

### 2.3 Trilineage differentiation of gADSCs

*In vitro* trilineage differentiation was performed to assess the multipotency of gADSCs, as described previously (Abraham et al., 2024; Abraham and Goel, 2025). Cells were seeded at 1 × 10^4^ cells/well in 12-well plates and cultured in lineage-specific differentiation media for adipogenic, chondrogenic, and osteogenic induction. Negative controls were maintained in complete medium without induction supplements. At the end of the induction period, cells were fixed with 4% paraformaldehyde and stained with Oil Red O, Alizarin Red, or Alcian Blue to evaluate adipogenic, osteogenic, or chondrogenic differentiation, respectively. Cells were examined using an inverted microscope, and images were acquired using a Michrome camera.

### 2.4 Culture of gADSCs in different oxygen concentrations for transcriptome analysis

**FIGURE 1:**
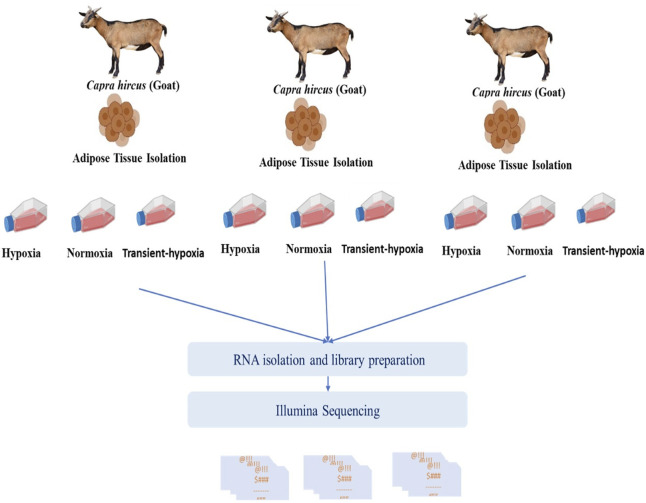
Schematic representation of the gADSC transcriptome data-analysis workflow.

For transcriptome analysis, donor-derived gADSC cultures were maintained independently and were not pooled. Normoxic cultures (NO) were maintained at 21% O_2_ and 5% CO_2_ for 72 h before harvesting. Transient hypoxia (THO) was defined as exposure to 2% O_2_ and 5% CO_2_ for 72 h before harvesting. Sustained hypoxia (HO) was defined as continuous culture at 2% O_2_ and 5% CO_2_ for approximately three passages prior to harvest. During routine passaging of HO cultures, cells were briefly handled under atmospheric oxygen for approximately 15–20 min for trypsinisation, counting, and reseeding, after which they were returned to the 2% O_2_ environment. Thus, HO cultures experienced repeated short reoxygenation intervals during passaging, whereas THO and NO cultures were harvested after a single 72 h exposure period. Cells used for RNA-seq were collected across comparable passage ranges whenever feasible, and the passage number was recorded for each donor-derived culture. Because sustained hypoxia also involves prolonged culture adaptation and brief intermittent reoxygenation during passaging, HO vs. THO comparisons were interpreted as differences between sustained/intermittently passaged hypoxic adaptation and transient hypoxic exposure rather than as a controlled comparison of oxygen tension alone. gADSC samples from three independent goat donors were harvested separately for RNA sequencing. Cell pellets were suspended in RNAlater and stored at −80 °C prior to shipment on dry ice to Nucleome Informatics Private Limited in Hyderabad, India. Figure 1 shows the schematic of the goat ADSC transcriptome data analysis performed.

### 2.5 RNA extraction and RNA sequencing

Total RNA was isolated from gADSCs using the MN NucleoSpin RNA Kit according to the manufacturer’s protocol. Briefly, harvested gADSCs were lysed using lysis buffer, and the lysates were sequentially processed through a DNA removal column, followed by an RNA binding column, to ensure complete removal of genomic DNA contamination. The RNA-binding column was washed with designated wash buffers and eluted with RNase-free water to obtain high-quality RNA suitable for cDNA synthesis. RNA integrity was assessed via 1% agarose gel electrophoresis (Lonza, Belgium), and the concentration was determined using a NanoDrop spectrophotometer. Only RNA samples with an RNA integrity number (RIN) > 8 were selected for sequencing. Library preparation was performed for all qualified RNA samples, and sequencing was conducted on the Illumina NovaSeq 6000 platform.

### 2.6 Bioinformatics data processing and analysis

The quality of sequenced reads was assessed using FastQC (Andrews, 2010) and summarised using MultiQC v1.34 (Ewels et al., 2016). Adapter sequences, low-quality bases, duplicated reads, and potential contaminants were removed using fastp v0.23.2 with default settings (Chen et al., 2018). High-quality reads were aligned to the *Capra hircus* ARS1.2 reference genome (GCF_001704415.2) using STAR v2.7.3a with a two-pass mapping strategy (Dobin et al., 2013). Gene-level read counts were generated using featureCounts v2.0.0 (Liao et al., 2014) based on the *Capra hircus* ARS1.2 genome annotation (National Centre for Biotechnology Information, 2016). Genes with an average read count <10 across samples were excluded from downstream analysis. Differential expression analysis was performed using DESeq2 v1.46.0 in R v4.4.2/Bioconductor (Love et al., 2014). Genes were considered statistically significant at an adjusted P value <0.05, and genes with |log2 fold-change| ≥1 were prioritised for downstream Gene Ontology (GO), Kyoto Encyclopedia of Genes and Genomes (KEGG), and protein-protein interaction (PPI) interpretation. Principal component analysis (PCA), sample-to-sample distance clustering, MA plots, and volcano plots were generated to evaluate replicate structure and visualise differential expression patterns. GO and KEGG enrichment analyses were performed using clusterProfiler (Yu et al., 2012). Venn diagrams were generated using Venny (Oliveros, 2007). PPI analysis was performed using STRING with a confidence score of 0.4 for *Capra hircus*, and hub/bottleneck genes were prioritised in Cytoscape using the cytoHubba plugin with the Maximal Clique Centrality (MCC) algorithm (Szklarczyk et al., 2023; Chin et al., 2014). Hub genes were defined as highly connected nodes, whereas bottleneck genes were defined as nodes with high betweenness centrality. Because goat interactome annotation is less complete than human or mouse interactomes, PPI-based prioritisation was interpreted as hypothesis-generating.

### 2.7 Validation of selected differentially expressed genes by quantitative reverse transcription PCR

Quantitative reverse transcription PCR (RT-qPCR) was performed to validate selected differentially expressed genes (DEGs) identified in the RNA-seq analysis and to assess their expression in gADSCs across NO, HO, and THO. Eight candidate oxygen-responsive genes were selected for validation: carbonic anhydrase 12 (*CA12*), calreticulin (*CALR*), dipeptidyl peptidase 4 (*DPP4*), growth arrest and DNA damage-inducible beta (*GADD45B*), poly (ADP-ribose) polymerase (*PARP*), snail family transcriptional repressor 1 (*SNAI1*), vascular endothelial growth factor A (*VEGFA*), and Wnt inhibitory factor 1 (*WIF1*). Five hub/bottleneck-associated genes, *RAD51* recombinase (*RAD51*), *BRCA1* DNA repair-associated (*BRCA1*), mitotic arrest deficient 2-like 1 (*MAD2L1*), thrombospondin 1 (*THBS1*), and hypoxia-inducible factor 1-alpha (*HIF1A*) were also validated. *HIF2A* was additionally included in the RT-qPCR validation panel, although it was not identified as a hub/bottleneck gene, because HIF-2α is a key regulator of sustained hypoxic adaptation and may functionally complement or diverge from HIF-1α-mediated responses. Its inclusion allowed comparison of *HIF1A* and *HIF2A* expression patterns across NO, HO, and THO conditions.

Total RNA was isolated from gADSCs cultured under NO, HO, and THO conditions and reverse-transcribed into complementary DNA (cDNA). RT-qPCR reactions were performed using TaKaRa SYBR Premix Ex Taq II on a QuantStudio 6 real-time PCR system. Primer sequences, annealing temperatures, and accession numbers are provided in Supplementary Material S1, Supplementary Table S1. Because reference gene expression may vary under altered oxygen tension, two housekeeping genes, beta-actin (*ACTB*) and ribosomal protein lateral stalk subunit P0 (*RPLP0*), were used for normalisation. Their use was based on consistent amplification across the three culture conditions; however, a formal geNorm/NormFinder-based reference gene stability analysis was not performed and is acknowledged as a limitation of the present study. Relative transcript abundance was calculated using the 2^−ΔΔCT^ method. The analysis included three independent biological replicates, with technical replicates for each reaction. Statistical analysis was performed using JMP software. Data are presented as mean ± SEM, and *P* < 0.05 was considered statistically significant.

## 3 Results

### 3.1 Isolation, culture, and characterisation of gADSCs

**FIGURE 2:**
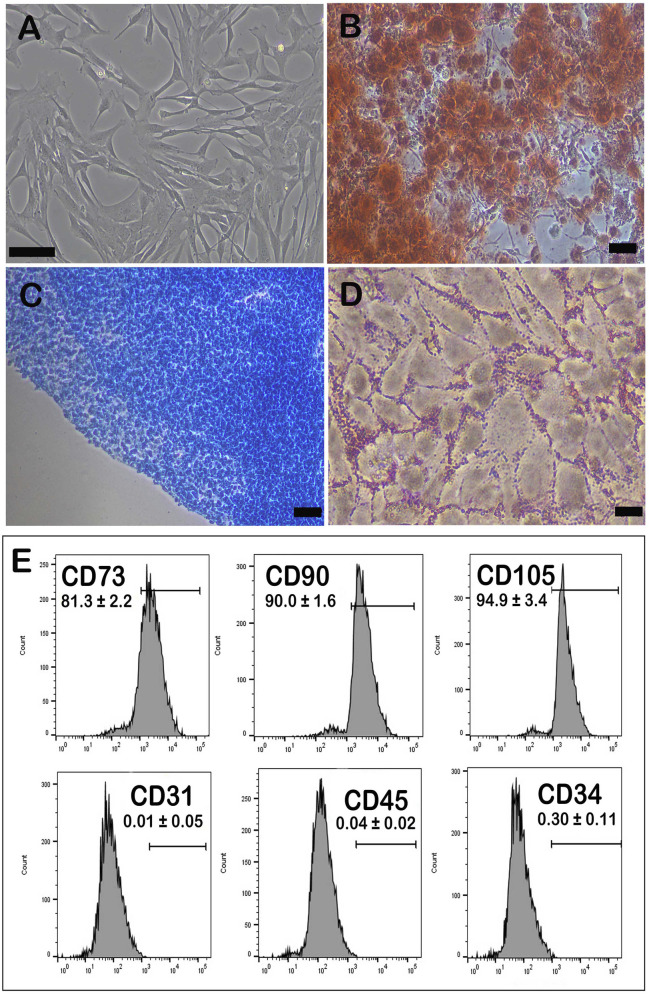
Isolation, differentiation, and immunophenotypic characterisation of gADSCs. **(A)** Phase-contrast image of sub-confluent goat adipose tissue-derived mesenchymal stem cells (gADSCs) at passage 4, showing typical fibroblast-like/spindle-shaped mesenchymal morphology. Trilineage differentiation potential of gADSCs was confirmed by lineage-specific staining: **(B)** osteogenic differentiation showing calcium deposition by Alizarin Red staining; **(C)** chondrogenic differentiation showing glycosaminoglycan-rich matrix by Alcian Blue staining; and **(D)** adipogenic differentiation showing intracellular lipid accumulation by Oil Red O staining. **(E)** Flow cytometric immunophenotyping of gADSCs showing high expression of mesenchymal stromal cell-associated markers CD73, CD90, and CD105, and minimal expression of hematopoietic/endothelial markers CD31, CD45, and CD34. Solid histograms represent marker-specific staining. Data shown for surface-marker expression represent mean percentage ±SEM from gADSCs derived from three independent donors and analysed between passages P6–P9. Scale bar = 40 µm.

The gADSCs were successfully isolated from inguinal adipose tissue collected from freshly slaughtered goats. The initial yield was 3.1 ± 0.8 × 10^5^ cells per gram of adipose tissue, with high post-isolation viability of 96.3% ± 3.1%. Following seeding at 2 × 10^4^ cells/cm in DMEM/F12 supplemented with 10% FBS, the cells adhered to the culture surface and exhibited progressive morphological adaptation. During early culture, the cells displayed polygonal, globular, or strip-like morphologies; however, upon successive passaging, they acquired a spindle-shaped, fibroblast-like morphology typical of MSCs (Figure 2A). The cultures showed robust expansion without overt morphological evidence of cellular stress, such as cytoplasmic vacuolation, membrane blebbing, or cell atrophy.

The multipotency of gADSCs was confirmed by trilineage differentiation assays. Under osteogenic induction, cells exhibited calcium deposition, as demonstrated by Alizarin Red staining (Figure 2B). Chondrogenic differentiation was confirmed by Alcian Blue staining, indicating glycosaminoglycan accumulation (Figure 2C). Adipogenic differentiation was demonstrated by Oil Red O-positive intracellular lipid droplets (Figure 2D). In contrast, cells maintained in complete medium without lineage-specific induction did not show lineage-associated staining, confirming the specificity of differentiation.

Flow cytometric immunophenotyping further confirmed the mesenchymal identity and purity of the isolated gADSCs. The cells expressed high levels of MSC-associated surface markers, including CD73 (81.3% ± 2.2%), CD90 (90.0% ± 1.6%), and CD105 (94.9% ± 3.4%), whereas hematopoietic/endothelial markers CD31, CD45, and CD34 were minimally expressed (<0.01%–0.30%) (Figure 2E). Together, the adherence pattern, spindle-shaped morphology, trilineage differentiation potential, and immunophenotypic profile confirmed that the isolated cells fulfilled the expected characteristics of gADSCs and were suitable for downstream transcriptomic analysis.

### 3.2 RNA-seq data generation, quality assessment, and global gene-expression overview

RNA sequencing was performed to examine transcriptional regulation in gADSCs cultured under NO, HO, and THO. A total of nine RNA-seq libraries, representing three independent biological replicates per oxygen condition, were generated according to the experimental workflow shown in Figure 1. Across all samples, 245.5 million raw reads were obtained, with approximately 93% of bases showing Q30 quality. After quality filtering, 204 million reads were retained, with approximately 95% Q30 bases. Following ribosomal RNA removal, 186.7 million high-quality reads were mapped to the *Capra hircus* ARS1.2 reference genome, with an average mapping rate of approximately 95% (Table 1). These quality metrics indicated that the sequencing data were suitable for downstream differential gene-expression analysis.

**TABLE 1:** A summary of generated sequencing data before and after quality control (QC) and mapping % of the over reference genome *Capra hircus* ARS 1.2 under three environmental conditions: sustained hypoxia (HO), transient hypoxia (THO), and normoxia (NO).

Serial number	Conditions	Before QC		After QC		Read count after rRNA removal (millions)	Read mapping (%)
		Reads (millions)	Q30 (%)	Reads (millions)	Q30 (%)		
1	HO	20.7	93.45	18.1	95.12	14.5	97.77
2	HO	25	93.4	19.3	95.17	18.1	95.65
3	HO	32.1	93.57	27	95.21	24.1	95.05
4	NO	29.4	93.62	25.7	95.3	24.2	95.99
5	NO	28	93.6	24.1	95.3	22.8	96.05
6	NO	29	93.43	23.4	95.26	21.6	97.77
7	THO	27	93.46	23	95.^16^	20.3	95.03
8	THO	26.6	93.68	20.7	95.33	19.3	94.39
9	THO	27.8	92.68	23.4	94.53	21.8	96.21

**FIGURE 3:**
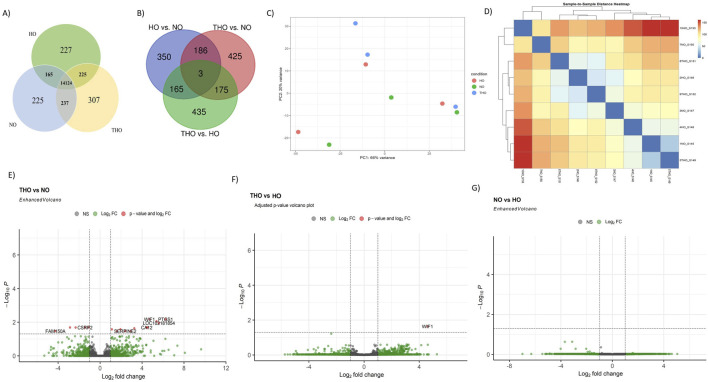
RNA-seq quality control and gene expression overview of gADSCs cultured under varying oxygen conditions. **(A)** Venn diagram showing the number of expressed genes detected under sustained hypoxia (HO), normoxia (NO), and transient hypoxia (THO), including shared and condition-specific expressed genes. A total of 14,124 genes were commonly expressed across all three conditions, whereas 227, 225, and 307 genes were uniquely expressed under HO, NO, and THO, respectively. **(B)** A Venn diagram showing the distribution of differentially expressed genes (DEGs) identified in the three pairwise comparisons: HO vs. NO, THO vs. NO, and THO vs. HO. The analysis revealed both comparison-specific and shared DEGs, with only three DEGs common to all comparisons. **(C)** Principal component analysis (PCA) plot showing the clustering pattern of biological replicates across oxygen conditions. **(D)** Sample-to-sample distance heatmap showing relationships among biological replicates based on global gene-expression profiles, with hierarchical clustering illustrating similarities and differences among samples. **(E–G)** Volcano plots showing differential gene expression for each pairwise comparison: **(E)** THO vs. NO, **(F)** THO vs. HO, and **(G)** NO vs. HO. Each point represents an individual gene. Grey points indicate non-significant genes, green points indicate genes passing the log_2_ fold-change threshold, and red points indicate genes passing both log_2_ fold-change and adjusted significance thresholds. The x-axis represents log_2_ fold-change, and the y-axis represents −log_10_ adjusted P value. Selected significant and/or biologically relevant genes are labelled. MA plots for the three comparisons are provided in the Supplementary Figure S2.

Gene expression was quantified across all three oxygen conditions after filtering low-expressed genes using an average read count threshold of ≥10. A total of 14,741, 14,893, and 14,751 genes were detected in HO, THO, and NO conditions, respectively. Among these, 14,124 genes were commonly expressed across all three oxygen conditions, whereas 227, 225, and 307 genes were uniquely expressed under HO, NO, and THO, respectively (Figure 3A; Supplementary Material S1; Supplementary Tables S2–S4). These findings indicate that although most genes were shared across culture conditions, each oxygen environment was associated with a distinct subset of condition-specific expressed genes.

Pairwise comparison of differentially expressed genes (DEGs) revealed both shared and condition-specific transcriptional responses. The HO vs. NO, THO vs. HO, and THO vs. NO comparisons contained 350, 435, and 425 unique DEGs, respectively. In addition, 186 DEGs were shared between HO vs. NO and THO vs. NO, 165 DEGs were shared between HO vs. NO and THO vs. HO, and 175 DEGs were shared between THO vs. NO and THO vs. HO. Only three DEGs were common to all three pairwise comparisons (Figure 3B). These data suggest that sustained and transient hypoxic exposures induce partially overlapping but distinct transcriptional programs in gADSCs.

Principal component analysis (PCA) showed partial separation of samples by oxygen condition, indicating that oxygen exposure contributed to global transcriptomic variation among the groups (Figure 3C). The first principal component accounted for 66% of the total variance, suggesting that treatment-associated transcriptional differences represented the major source of variation in the dataset. The sample-to-sample distance heatmap further supported treatment-related clustering and showed detectable inter-sample variation, consistent with donor-level biological variability among independently derived gADSC cultures (Figure 3D).

Volcano plot analysis further illustrated condition-dependent differential gene expression across the three pairwise comparisons (Figures 3E–G). In the THO vs. NO comparison, several genes showed pronounced fold-change differences, including both upregulated and downregulated transcripts (Figure 3E). The THO vs. HO comparison revealed transcriptional changes associated with the transition from transient to sustained hypoxic exposure, although fewer genes met the combined fold-change and statistical significance thresholds (Figure 3F). In contrast, the NO vs. HO comparison showed a relatively compressed distribution of significant transcripts, indicating that a subset of genes changed substantially between normoxic and sustained-hypoxic culture conditions (Figure 3G). MA plots for the three comparisons are provided in the Supplementary Material S3 (Supplementary Figure S1A). The MA plots demonstrate that oxygen exposure and exposure duration modulate gene expression in gADSCs, with most genes remaining stable and a defined subset showing condition-dependent differential regulation. Collectively, these analyses confirmed acceptable RNA-seq data structure and demonstrated that NO, HO, and THO conditions induce both shared and distinct transcriptomic responses in gADSCs.

### 3.3 Differential gene-expression analysis

Differential gene-expression analysis was performed for the three pairwise comparisons: HO vs. NO, THO vs. HO, and THO vs. NO. Genes were considered differentially expressed using an adjusted P value <0.05, and genes with |log2 fold-change| >=1 were prioritised for biological interpretation and downstream enrichment/PPI analyses. Overall, 704 DEGs were identified in HO vs. NO, including 399 upregulated and 305 downregulated genes. In the HO vs. THO comparison, 778 DEGs were detected, of which 450 were upregulated, and 328 were downregulated. The THO vs. NO comparison yielded 789 DEGs, including 361 upregulated and 428 downregulated genes (Table 2). Complete DEG statistics, including Ensembl gene ID, gene name, log2 fold-change, raw P value, and adjusted P value, are provided in Supplementary Material S2.

**TABLE 2:** Summary of differential gene expression analysis for *Capra hircus* under sustained hypoxia (HO), transient hypoxia (THO), and normoxia (NO). Genes were considered statistically significant at adjusted P < 0.05; genes with |log2 fold-change| >=1 were prioritised for downstream biological interpretation.

Comparison	Condition	Total DEGs	Upregulated genes	Downregulated genes
1	HO vs. NO	704	399	305
2	HO vs. THO	778	450	328
3	THO vs. NO	789	361	428

The top upregulated and downregulated genes for each comparison are summarised in Table 3. In the HO vs. NO comparison, prostaglandin-endoperoxide synthase 1 (*PTGS1*; log_2_FC = 2.485, adjusted P = 0.0470) and carbonic anhydrase 12 (*CA12*; log_2_FC = 2.380, adjusted P = 0.0196) were among the top upregulated genes, whereas glutaminyl-peptide cyclotransferase-like protein (*QPCTL*; log_2_FC = −1.059, adjusted P = 0.0041) was among the downregulated genes (Table 3). In the HO vs. THO comparison, adenylate cyclase-activating polypeptide 1 (*ADCYAP1*; log_2_FC = 3.884, adjusted P = 0.0061) and IQ motif and ankyrin repeat containing 1 (*IQANK1*; log_2_FC = 3.533, adjusted P = 0.0237) were among the most upregulated genes, whereas Ras protein-specific guanine nucleotide-releasing factor 2 (*RASGRF2*; log_2_FC = −2.690, adjusted P = 0.0270) was among the downregulated genes (Table 3). In the THO vs. NO comparison, coiled-coil domain-containing protein 1 (*CABCOCO1*; log_2_FC = 1.207, adjusted P = 0.0281), macroH2A.2 histone (*MACROH2A2*; log_2_FC = 3.383, adjusted P = 0.0140), and *RAB31* member RAS oncogene family (*RAB31*; log_2_FC = 0.676, adjusted P = 0.0271) were among the upregulated genes, whereas interferon-related developmental regulator 2 (*IFRD2*; log_2_FC = −1.192, adjusted P = 0.00551) and *QPCTL* (log_2_FC = −0.757, adjusted P = 0.0408) were downregulated (Table 3). These findings indicate that both sustained and transient hypoxia remodel the gADSC transcriptome, with THO showing the largest number of DEGs compared with NO.

**TABLE 3:** Summary of the top three upregulated and downregulated genes (Ensembl gene ID, gene name, log2FC, and adjusted P value) for each comparison.

Group	Ensemble gene ID	Gene name	Log2FC	Adj. P	Differentially expressed
HO Vs. NO	ENSCHIG00000000110	NA	0.982769	0.0437	UP
	ENSCHIG00000000527	*PTGS1*	2.485175	0.0470	UP
	ENSCHIG00000000239	*CA12*	2.379669	0.0196	UP
	ENSCHIG00000027222	*QPCTL*	−1.05876	0.0041	DOWN
	ENSCHIG00000027233	NA	−0.90154	0.014	DOWN
	ENSCHIG00000027244	NA	−0.52753	0.0310	DOWN
HO Vs. THO	ENSCHIG00000000123	*IQANK1*	3.533018	0.0237	UP
	ENSCHIG00000000212	*ADCYAP1*	3.884187	0.0061	UP
	ENSCHIG00000000253	NA	6.128597	0.026	UP
	ENSCHIG00000027194	*RASGRF2*	−2.69007	0.027	DOWN
	ENSCHIG00000027002	NA	−1.41311	0.0109	DOWN
	ENSCHIG00000027224	NA	−1.38355	0.0277	DOWN
THO Vs. NO	ENSCHIG00000000104	*CABCOCO1*	1.206969	0.0281	UP
	ENSCHIG00000000171	*MACROH2A2*	3.383307	0.014	UP
	ENSCHIG00000000146	*RAB31*	0.675963	0.0271	UP
	ENSCHIG00000027019	*IFRD2*	−1.19247	0.00551	DOWN
	ENSCHIG00000027214	NA	−0.76855	0.0497	DOWN
	ENSCHIG00000027222	*QPCTL*	−0.75699	0.0408	DOWN

### 3.4 Gene Ontology and KEGG pathway enrichment analyses

Gene Ontology (GO) enrichment analysis was performed separately for unique and shared DEGs to identify biological processes associated with oxygen-dependent transcriptional remodelling. Among unique DEGs, no significantly enriched GO term was detected in the HO vs. NO comparison (Table 4). In contrast, unique DEGs in the THO vs. NO comparison were significantly enriched for mitotic cell cycle process (significance = 1.35E−11), DNA-templated DNA replication (7.42E−05), base-excision repair (1.32E−04), organic cyclic compound biosynthetic process (0.00213), and sterol metabolic process (0.02891) (Table 4). These enrichments indicate that transient hypoxia relative to normoxia strongly affected genes associated with cell-cycle progression, DNA replication, DNA repair, and metabolic regulation.

**TABLE 4:** GO analysis of unique treatment-associated DEGs and their key biological processes.

Serial number	Experiment	Biological process	Significance
1	HO vs. NO	NA	NA
1	THO vs. NO	Mitotic cell cycle process	1.35E-11
2		DNA-templated DNA replication	7.41606E-05
3		Base-excision repair	0.000132273
4		Organic cyclic compound biosynthetic process	0.002133505
5		Sterol metabolic process	0.028907625
1	THO Vs. HO	Cell motility	5.56E
2		Tissue remodelling	2.77376E-05
3		Extracellular matrix organisation	3.64732E-05
4		Response to stimulus	7.02807E-05
5		Elastin metabolic process	0.001077293
6		Wound healing	0.004606985
7		Actomyosin structure organisation	0.004618532
8		Response to oxygen-containing compound	0.015557002
9		Inflammatory response	0.024921343
10		Regulation of protein metabolic process	0.04277439
11		Growth	0.049207204

Unique DEGs in the THO vs. HO comparison were enriched for several processes related to cell movement, extracellular structure, and stress response. These included cell motility, tissue remodelling (2.77E−05), extracellular matrix organisation (3.65E−05), response to stimulus (7.03E−05), elastin metabolic process (0.00108), wound healing (0.00461), actomyosin structure organisation (0.00462), response to oxygen-containing compound (0.01556), inflammatory response (0.02492), regulation of protein metabolic process (0.04277), and growth (0.04921) (Table 4). These results suggest that the transition between transient and sustained hypoxia is associated with changes in extracellular matrix remodelling, inflammatory signalling, cytoskeletal organisation, and oxygen-responsive stress pathways.

GO analysis of shared DEGs revealed additional biological processes common across pairwise comparisons. DEGs shared between NO vs. HO and NO vs. THO were enriched for cell cycle process (4.65E−40), protein localisation to the chromosome centromeric region (2.48E−05), DNA metabolic process (3.06E−05), DNA damage response (0.00491), regulation of ubiquitin-protein ligase activity (0.00954), protein modification process, protein localisation to the microtubule cytoskeleton, and positive regulation of nitrogen compound metabolic process (Table 5). Shared DEGs between HO vs. NO and HO vs. THO were enriched for temperature homeostasis and regulation of response to stimulus (Table 5). In contrast, DEGs shared between THO vs. NO and THO vs. HO were enriched for regulation of developmental processes (4.96E−10), positive regulation of supramolecular fibre organisation (0.000231), muscle system processes, cell-substrate adhesion, endothelial cell development, and cell surface receptor signalling pathways (Table 5). Collectively, these results indicate that oxygen conditions and exposure duration influence gADSC processes related to cell-cycle control, DNA damage response, cytoskeletal organisation, cell adhesion, and developmental regulation.

**TABLE 5:** GO analysis of identified shared DEGs for treatment, and their associated biological process.

Serial number	Experiment	Biological process	Significance
1	NO vs. HO NO vs. THO	Cell cycle process	4.65E-40
2		Protein localisation to the chromosome, centromeric region	2.4788E-05
3		DNA metabolic process	3.06159E-05
4		DNA damage response	0.00491141
5		Regulation of ubiquitin-protein ligase activity	0.009535064
6		Positive regulation of the biological process	0.034616492
7		Protein modification process	0.037533943
8		Protein localisation to the microtubule cytoskeleton	0.046005384
9		Positive regulation of nitrogen compound metabolic process	0.04674092
1	HO vs. NO HO vs. THO	Temperature homeostasis	0.030777381
2		Regulation of response to stimulus	0.046581275
1	THO vs. NO THO vs. HO	Regulation of the developmental process	4.96E-10
2		Positive regulations of supramolecular fibre organisation	0.000231118
3		Muscle system process	0.023498372
4		Cell-substrate adhesion	0.033535677
5		Endothelial cell development	0.035867298
6		Cell surface receptor signalling pathway	0.043802204

**FIGURE 4:**
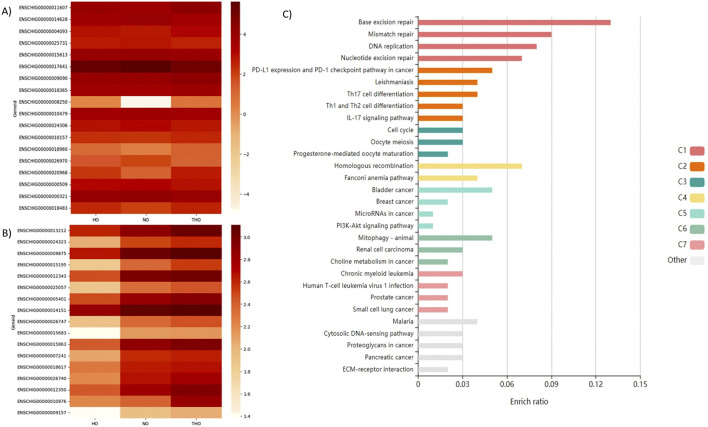
Hub and bottleneck gene expression and pathway enrichment analysis. **(A,B)** Heatmaps showing the expression profiles of identified hub and bottleneck genes across hypoxia (HO), normoxia (NO), and transient hypoxia (THO) conditions. Genes are represented by Ensembl gene IDs, and the colour intensity indicates relative expression levels, with darker red representing higher expression and lighter shades representing lower expression. **(C)** KEGG pathway enrichment analysis of unique hub and bottleneck genes. The x-axis represents the enrichment ratio, and pathways are grouped into major functional clusters, with pathways within the same cluster shown in the same colour. C1 represents DNA repair and genome maintenance pathways, including base excision repair, mismatch repair, DNA replication, and nucleotide excision repair. C2 represents immune and inflammatory signalling pathways, including PD-L1 expression and PD-1 checkpoint pathway in cancer, leishmaniasis, Th17 cell differentiation, Th1 and Th2 cell differentiation, and IL-17 signalling. C3 represents cell-cycle and reproductive maturation-associated pathways, including cell cycle, oocyte meiosis, and progesterone-mediated oocyte maturation. C4 represents DNA damage repair and chromosomal stability pathways, including homologous recombination and the Fanconi anaemia pathway. C5 represents cancer-associated signalling pathways, including bladder cancer, breast cancer, microRNAs in cancer, and PI3K–Akt signalling pathway. C6 represents mitochondrial quality control, metabolism, and cancer-related pathways, including mitophagy, renal cell carcinoma, and choline metabolism. C7 represents haematological malignancy, viral infection, and tumour-associated pathways, including chronic myeloid leukaemia, human T-cell leukaemia virus 1 infection, prostate cancer, and small cell lung cancer. Pathways classified as Other include malaria, the cytosolic DNA-sensing pathway, proteoglycans in cancer, pancreatic cancer, and ECM–receptor interaction.

Kyoto Encyclopedia of Genes and Genomes (KEGG) enrichment analysis further supported the GO findings. Enriched pathways among unique DEGs were associated with DNA replication, cell cycle regulation, immune-cell differentiation, infection and immune activation, cancer-associated signalling, and homologous recombination. These pathways indicate that oxygen transitions in gADSCs are associated with coordinated transcriptional programs related to cell stress, DNA repair, proliferative regulation, immune/inflammatory signalling, and genome maintenance (Figure 4C).

### 3.5 Protein-protein interaction analysis and prioritisation of hub and bottleneck genes

Protein-protein interaction (PPI) network analysis was performed to visualise the topological relationships among hub and bottleneck genes identified from unique differentially expressed genes (DEGs) in the transient hypoxia versus normoxia (THO vs. NO) and transient hypoxia versus sustained hypoxia (THO vs. HO) comparisons. In the THO vs. NO network, several genes involved in DNA repair, replication, and cell-cycle regulation occupied central positions within the interaction network (Supplementary Material S3; Supplementary Figures S1B,S1C). Notably, *RAD51*, *BRCA1*, and *MAD2L1* were identified as overlapping hub and bottleneck genes, suggesting their potential importance in coordinating DNA damage response, homologous recombination, and mitotic checkpoint-associated pathways during the transition from normoxia to transient hypoxia.

In the THO vs. HO comparison, the PPI network highlighted genes associated with hypoxia signalling, extracellular matrix remodelling, cell adhesion, inflammatory signalling, and stress-response pathways (Supplementary Figure S1B). *HIF1A* and *THBS1* appeared as overlapping hub and bottleneck genes, indicating their potential centrality in distinguishing transient hypoxic adaptation from sustained hypoxic conditioning. Together, these supplementary PPI networks support the prioritisation of key candidate regulatory genes for downstream validation and provide a network-level basis for interpreting oxygen-dependent transcriptional remodelling in gADSCs. The top ten hub and bottleneck genes identified from the relevant comparisons are presented in Tables 6, 7, respectively.

**TABLE 6:** List of the top ten hub genes in each unique treatment.

Experiment	Hub genes	Gene id	Gene description	Biological process
HO vs. NO	NA	NA	NA	NA
THO vs. NO	*RAD51*	ENSCHIG00000026747	*RAD51* recombinase	Homologous DNA pairing and strand exchange
	*BRCA1*	ENSCHIG00000026740	*BRCA1* DNA repair-associated	Homologous DNA pairing and strand exchange
	*NCAPG*	ENSCHIG00000012343	Non-SMC condensin	EML4 and NUDC in mitotic spindle formation
	*POLE*	ENSCHIG00000007241	DNA polymerase epsilon, catalytic subunit	Base excision repair
	*ESPL1*	ENSCHIG00000015195	Extra spindle pole bodies like 1, separase	Separation of sister chromatids
	*KIF20B*	ENSCHIG00000012350	Kinesin family member 20B	Vesicle-mediated transport
	*SMC2*	ENSCHIG00000005401	Structural maintenance of chromosome 2	Cell cycle, RNA polymerase I promoter opening
	*RACGAP1*	ENSCHIG00000009875	Rac GTPase-activating protein 1	Signalling by Rho GTPases
	*NEIL3*	ENSCHIG00000009157	Nei like DNA glycosylase 3	Homologous DNA pairing and strand exchange
	*MAD2L1*	ENSCHIG00000025057	Mitotic arrest deficient 2 like 1	EML4 and NUDC in mitotic spindle formation
THO vs. HO	*CST3*	ENSCHIG00000009090	Cystatin C	Innate immune system
	*IGFBP5*	ENSCHIG00000011607	Insulin-like growth factor binding protein 5	Regulation of IGF and IGFBPs
	*GPC3*	ENSCHIG00000018483	Glypican 3	Glycosaminoglycan metabolism
	*WFS1*	ENSCHIG00000025731	Wolframin ER transmembrane glycoprotein	Unfolded protein response Cellular response to stimuli
	*APOA5*	ENSCHIG00000008250	Apolipoprotein 5	Plasma lipoprotein assembly, remodelling and clearance
	*FAM20A*	ENSCHIG00000026970	*FAM20A* Golgi-associated secretory pathway	Amelogenesis imperfecta Hypoplastic type
	*THBS1*	ENSCHIG00000017641	Thrombospondin	Response to elevated platelet cytosolic Ca2+
	*HIF1A*	ENSCHIG00000015613	HO inducible factor 1	Tissue remodelling, wound healing
	*PECAM1*	ENSCHIG00000018960	Platelet and endothelial cell adhesion molecule 1	Response to elevated platelet cytosolic Ca2+ and innate immune system
	*ADAMTS6*	ENSCHIG00000024306	ADAM metallopeptidase with thrombospondin type 1 motif 6	Disease associated with O-glycosylation of proteins

**TABLE 7:** List of the top ten bottleneck genes in each unique treatment.

Experiment	Bottleneck genes	Gene id	Log2fold change	Gene description	Biological process
HO vs. NO	NA	NA	NA	NA	NA
THO vs. NO	*RAD51*	ENSCHIG00000026747	2.774007732	*RAD51* recombinase	Homologous DNA pairing and strand exchange
	*DTYMK*	ENSCHIG00000024323	1.757617285	Deoxythymidylate kinase	Pyrimidine metabolism
	*PCNA*	ENSCHIG00000015863	1.602388667	Proliferating cell nuclear antigen	Base excision repair
	*POLD1*	ENSCHIG00000018617	1.235181464	DNA polymerase delta 1, catalytic subunit	DNA repair pathways and base excision repair
	*LSS*	ENSCHIG00000013212	1.377340634	Lanosterol synthase	Super pathways of cholesterol biosynthesis and metabolism of steroids
	*BRCA1*	ENSCHIG00000026740	1.666416662	*BRCA1* DNA repair-associated	Homologous DNA pairing and strand exchange
	*MAD2L1*	ENSCHIG00000025057	1.869373922	Mitotic arrest deficient 2 like 1	EML4 and NUDC in mitotic spindle formation
	*POLR2E*	ENSCHIG00000014151	0.642618613	RNA polymerase II, I and III subunit E	Formation of the HIV elongation complex in the absence of HIV Tat
	*HMGA1*	ENSCHIG00000010976	1.908409426	High mobility group AT-hook 1	HIV life cycle and cellular response to stimuli
	*E2F1*	ENSCHIG00000015683	2.295411696	E2F transcription factor 1	Intrinsic pathways for apoptosis
THO vs. HO	*HIF1A*	ENSCHIG00000015613	−0.510637221	HO inducible factor 1	Tissue remodelling, wound healing
	*VCL*	ENSCHIG00000000321	−0.617902995	Vinculin	Signalling downstream of RAS mutants
	*THBS1*	ENSCHIG00000017641	−0.967073344	Thrombospondin 1	Response to elevated platelet cytosolic Ca2+
	*MAPK14*	ENSCHIG00000000509	−0.408227777	Mitogen-activated protein kinase 14	Prolactin signalling
	*NFKBIA*	ENSCHIG00000010157	0.405633143	NFKB inhibitor alpha	TNFR1 pathway
	*JUN*	ENSCHIG00000004093	0.525788838	Jun proto-oncogene, AP-1 transcription factor subunit	Prolactin signalling
	*IGF2*	ENSCHIG00000020968	−0.33991	Insulin-like growth factor 2	Apoptotic pathways in synovial fibroblasts and the GPCR pathway
	*SDC4*	ENSCHIG00000018365	0.216787098	Syndecan 4	Glycosaminoglycan metabolism
	*TNS1*	ENSCHIG00000014628	−0.311987621	Tensin 1	Integrin-mediated cell adhesion
	*GNB1*	ENSCHIP00000006823	−0.12687688	G protein subunit beta 1	Thromboxane signalling through TP receptor

No hub or bottleneck gene was identified for the HO vs. NO comparison, consistent with the absence of significant GO enrichment among unique DEGs in this comparison (Tables 6, 7). In the THO vs. NO comparison, several hub genes were associated with DNA repair, mitotic regulation, and cell-cycle-associated processes. The top hub genes included *RAD51* recombinase (*RAD51*), *BRCA1* DNA repair associated (*BRCA1*), non-SMC condensin I complex subunit G (*NCAPG*), DNA polymerase epsilon catalytic subunit (*POLE*), extra spindle pole bodies like 1 (*ESPL1*), kinesin family member 20B (*KIF20B*), structural maintenance of chromosomes 2 (*SMC2*), Rac GTPase-activating protein 1 (*RACGAP1*), Nei-like DNA glycosylase 3 (*NEIL3*), and mitotic arrest deficient 2 like 1 (*MAD2L1*) (Table 6). In the same comparison, bottleneck genes included *RAD51*, deoxythymidylate kinase (*DTYMK*), proliferating cell nuclear antigen (*PCNA*), DNA polymerase delta 1 catalytic subunit (*POLD1*), lanosterol synthase (*LSS*), *BRCA1*, *MAD2L1*, RNA polymerase II, I and III subunit E (*POLR2E*), high mobility group AT-hook 1 (*HMGA1*), and E2F transcription factor 1 (*E2F1*) (Table 6). The overlap of *RAD51*, *BRCA1*, and *MAD2L1* between hub and bottleneck categories highlights a central network involving homologous recombination, DNA repair, and mitotic checkpoint control in THO vs. NO.

In the THO vs. HO comparison, the top hub genes included cystatin C (*CST3*), insulin-like growth factor binding protein 5 (*IGFBP5*), glypican 3 (*GPC3*), wolframin ER transmembrane glycoprotein (*WFS1*), apolipoprotein A5 (*APOA5*), *FAM20A* Golgi-associated secretory pathway kinase (*FAM20A*), thrombospondin 1 (*THBS1*), hypoxia-inducible factor 1-alpha (*HIF1A*), platelet and endothelial cell adhesion molecule 1 (*PECAM1*), and ADAM metallopeptidase with thrombospondin type 1 motif 6 (*ADAMTS6*) (Table 6). The corresponding bottleneck genes included *HIF1A*, vinculin (*VCL*), *THBS1*, mitogen-activated protein kinase 14 (*MAPK14*), NF-κB inhibitor alpha (*NFKBIA*), Jun proto-oncogene/AP-1 transcription factor subunit (*JUN*), insulin-like growth factor 2 (*IGF2*), syndecan 4 (*SDC4*), tensin 1 (*TNS1*), and G protein subunit beta 1 (*GNB1*) (Table 7). The shared prioritisation of *HIF1A* and *THBS1* as hub/bottleneck genes suggests that hypoxia signalling, extracellular matrix regulation, adhesion, and stress-responsive pathways contribute to transcriptional differences between transient and sustained hypoxic exposure.

Heatmap analysis of hub and bottleneck genes showed condition-associated expression patterns across HO, NO, and THO samples (Figures 4A,B). KEGG enrichment of unique hub and bottleneck genes further grouped enriched pathways into seven major functional clusters (Figure 4C). Cluster C1 comprised DNA repair and genome maintenance pathways, including base excision repair, mismatch repair, DNA replication, and nucleotide excision repair. Cluster C2 included immune and inflammatory signalling pathways, such as PD-L1 expression and PD-1 checkpoint pathway in cancer, leishmaniasis, Th17 cell differentiation, Th1/Th2 cell differentiation, and IL-17 signalling. Cluster C3 represented cell-cycle and reproductive maturation-related pathways, including cell cycle, oocyte meiosis, and progesterone-mediated oocyte maturation. Cluster C4 included DNA damage repair and chromosomal stability pathways, such as homologous recombination and the Fanconi anaemia pathway. Cluster C5 included cancer-associated signalling pathways, including bladder cancer, breast cancer, microRNAs in cancer, and PI3K–Akt signalling. Cluster C6 represented mitochondrial quality control, metabolic, and cancer-related pathways, including mitophagy, renal cell carcinoma, and choline metabolism. Cluster C7 included haematological malignancy, viral infection, and tumour-associated pathways, including chronic myeloid leukaemia, human T-cell leukaemia virus 1 infection, prostate cancer, and small cell lung cancer. Other enriched pathways included malaria, the cytosolic DNA-sensing pathway, proteoglycans in cancer, pancreatic cancer, and ECM–receptor interaction (Figure 4C). These findings indicate that hub and bottleneck genes converge on networks associated with DNA repair, cell-cycle regulation, immune signalling, extracellular matrix remodelling, and hypoxia-responsive adaptation.

### 3.6 RT-qPCR validation of selected oxygen-responsive DEGs

**FIGURE 5:**
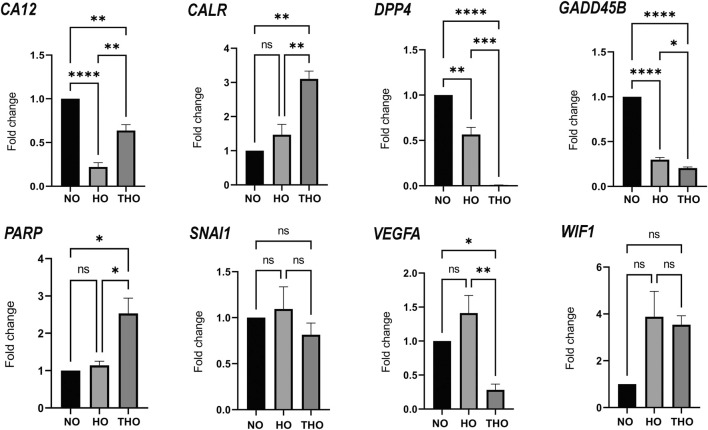
RT-qPCR validation of selected oxygen-responsive differentially expressed genes in gADSCs. Relative mRNA expression of selected differentially expressed genes was analysed in goat adipose tissue-derived mesenchymal stem cells (gADSCs) cultured under normoxia (NO), sustained hypoxia (HO), and transient hypoxia (THO). The validated genes included carbonic anhydrase 12 (CA12), calreticulin (CALR), dipeptidyl peptidase 4 (DPP4), growth arrest and DNA damage-inducible beta (GADD45B), poly (ADP-ribose) polymerase (PARP), snail family transcriptional repressor 1 (SNAI1), vascular endothelial growth factor A (VEGFA), and Wnt inhibitory factor 1 (WIF1). Gene-expression levels were normalised using two housekeeping genes, ACTB and RPLP0, and relative expression was calculated using the 2^−ΔΔCt method. Data are presented as mean ± SEM from three independent biological replicates with technical replicates for each reaction. Statistical significance is indicated as *P < 0.05, **P < 0.01, ***P < 0.001, and ****P < 0.0001; ns, not significant.

Eight candidate DEGs were selected for RT-qPCR validation across NO, HO, and THO conditions to confirm RNA-seq-derived expression patterns and evaluate oxygen-responsive transcriptional regulation in gADSCs. The selected genes included *CA12*, *CALR*, *DPP4*, *GADD45B*, *PARP*, *SNAI1*, *VEGFA*, and *WIF1* (Figure 5). *CA12* expression was significantly reduced in gADSCs cultured under HO and THO compared with NO. The decrease was more pronounced under HO (P < 0.0001) than under THO (P < 0.01), and *CA12* expression was also significantly lower in HO than in THO (P < 0.01). This pattern indicates oxygen-dependent suppression of *CA12*, with sustained hypoxia exerting the strongest effect. *CALR* expression showed a distinct transient hypoxia-associated increase. Although *CALR* expression under HO did not differ significantly from NO (P > 0.05), expression was significantly higher under THO than under both NO and HO (P < 0.01). This suggests that *CALR* may be preferentially induced during transient hypoxic adaptation. *DPP4* expression was significantly reduced under HO compared with NO (P < 0.01) and was nearly abolished under THO. Transcript levels under THO were significantly lower than those under both NO (P < 0.0001) and HO (P < 0.001), indicating strong oxygen-dependent repression of *DPP4*, particularly under transient hypoxia. *GADD45B* expression was significantly reduced in both HO and THO compared with NO (P < 0.0001). In addition, expression was further reduced under THO compared with HO (P < 0.05), suggesting that transient hypoxia may exert a stronger suppressive effect on this stress-response and DNA damage-associated gene. *PARP* expression was significantly increased under THO compared with both NO and HO (P < 0.05), whereas no significant difference was observed between NO and HO (P > 0.05). This expression pattern indicates preferential upregulation of *PARP* under transient hypoxia. *SNAI1* expression did not differ significantly among NO, HO, and THO conditions (P > 0.05), suggesting that *SNAI1* was not detectably modulated by the oxygen conditions tested in this study. Similarly, *WIF1* expression showed no statistically significant differences across the three oxygen conditions (P > 0.05), although it was numerically higher under HO and THO than under NO. *VEGFA* expression showed a condition-specific pattern. Although HO showed a numerical increase relative to NO, the difference was not statistically significant (P > 0.05). In contrast, *VEGFA* expression was significantly reduced under THO compared with NO (P < 0.05) and HO (P < 0.01), indicating that transient hypoxia was associated with suppression of *VEGFA* transcript abundance in gADSCs. To directly compare RNA-seq-derived expression patterns with RT-qPCR validation outcomes, the direction and significance of expression changes for validated genes were summarised side by side (Table 8). This comparison showed that several genes displayed concordant or partially concordant regulation across platforms, whereas *CA12*, *SNAI1*, *WIF1*, and *VEGFA* showed discordant or non-significant validation patterns, indicating that transcriptomic findings for these genes should be interpreted with caution.

**TABLE 8:** Concordance between RNA-seq-derived expression patterns and RT-qPCR validation of selected genes.

Gene	Selection basis	RNA-seq comparison	RNA-seq direction and magnitude	RT-qPCR direction and magnitude	RT-qPCR significance	Concordance status	Interpretation
*CA12*	DEG selected for validation	HO vs. NO	Upregulated; log_2_FC = 2.38, adj. P = 0.0196	Downregulated under HO and THO relative to NO	HO vs. NO: ****; THO vs. NO: **; HO vs. THO: **	Discordant	RNA-seq and RT-qPCR showed opposite directionality. This gene should be interpreted cautiously and requires validation using independent primers, isoform-specific analysis, and protein/functional assays
*CALR*	DEG selected for validation	RNA-seq-selected candidate	Direction/magnitude available in Supplementary Material S2	Increased under THO relative to NO and HO	THO vs. NO: **; THO vs. HO: **; HO vs. NO: ns	Partially concordant/validated by RT-qPCR	RT-qPCR supports oxygen-associated regulation, particularly transient hypoxia-associated induction
*DPP4*	DEG selected for validation	RNA-seq-selected candidate	Direction/magnitude available in Supplementary Material S2	Reduced under HO and strongly reduced under THO relative to NO	HO vs. NO: **; THO vs. NO: ****; THO vs. HO: ***	Concordant/validated by RT-qPCR	RT-qPCR supports oxygen-associated suppression, with the strongest effect under THO.
*GADD45B*	DEG selected for validation	RNA-seq-selected candidate	Direction/magnitude available in Supplementary Material S2	Reduced under HO and THO relative to NO	HO vs. NO: ****; THO vs. NO: ****; THO vs. HO: *	Concordant/validated by RT-qPCR	RT-qPCR supports oxygen-associated suppression of this stress-response transcript
*PARP*	DEG selected for validation	RNA-seq-selected candidate	Direction/magnitude available in Supplementary Material S2	Increased under THO relative to NO and HO	THO vs. NO: *; THO vs. HO: *; HO vs. NO: ns	Partially concordant/validated by RT-qPCR	RT-qPCR supports preferential THO-associated induction
*SNAI1*	DEG selected for validation	RNA-seq-selected candidate	Direction/magnitude available in Supplementary Material S2	No significant change among NO, HO, and THO	ns	Not validated	RT-qPCR did not confirm oxygen-dependent modulation; it should not be interpreted as an oxygen-responsive gene under the present conditions
*VEGFA*	DEG selected for validation; angiogenesis-related gene	RNA-seq-selected candidate	Direction/magnitude available in Supplementary Material S2	Reduced under THO relative to NO and HO; HO not significantly different from NO	THO vs. NO: *; THO vs. HO: **; HO vs. NO: ns	Partially concordant/unexpected	The THO-associated reduction is unexpected and should be interpreted as a transcript-level observation that requires VEGF-A protein and endothelial functional validation
*WIF1*	DEG selected for validation	RNA-seq-selected candidate	Direction/magnitude available in Supplementary Material S2	No significant change among NO, HO, and THO	ns	Not validated	RT-qPCR did not confirm significant oxygen-dependent regulation
*RAD51*	Hub/bottleneck gene	THO vs. NO; THO vs. HO network	Bottleneck log_2_FC = 2.77 in Table 7	Reduced under HO and THO relative to NO	HO vs. NO: ****; THO vs. NO: ****; HO vs. THO: ****	Directionally discordant with the listed network fold-change/validated as oxygen-responsive	RT-qPCR supports oxygen-associated suppression; RNA-seq/qRT-PCR directionality should be interpreted with caution if based on a different comparison orientation
*BRCA1*	Hub/bottleneck gene	THO vs. NO network	Bottleneck log_2_FC = 1.67 in Table 7	Reduced under HO and THO relative to NO	HO vs. NO: ****; THO vs. NO: ****; HO vs. THO: **	Directionally discordant with the listed network fold-change/validated as oxygen-responsive	RT-qPCR supports oxygen-associated suppression of DNA repair-associated transcripts
*MAD2L1*	Hub/bottleneck gene	THO vs. NO network	Bottleneck log_2_FC = 1.87 in Table 7	Reduced under HO and THO relative to NO	HO vs. NO: ***; THO vs. NO: ****; THO vs. HO: *	Directionally discordant with the listed network fold-change/validated as oxygen-responsive	RT-qPCR supports oxygen-associated modulation of mitotic checkpoint-associated transcripts
*THBS1*	Hub/bottleneck gene	THO vs. HO network	Bottleneck log_2_FC = −0.97 in Table 7	Strongly increased under HO and THO relative to NO	HO vs. NO: ***; THO vs. NO: ***; HO vs. THO: ns	Partially concordant/comparison-dependent	RT-qPCR indicates strong hypoxia-associated induction, but RNA-seq comparison orientation should be considered
*HIF1A*	Hub/bottleneck gene	THO vs. HO network	Bottleneck log_2_FC = −0.51 in Table 7	Reduced under HO and THO relative to NO	HO vs. NO: ****; THO vs. NO: ***; HO vs. THO: ***	Partially concordant/validated as oxygen-responsive	RT-qPCR supports differential transcript regulation, but HIF-1α signalling requires protein-level validation
*HIF2A*	Hypothesis-driven HIF isoform comparator	Not hub/bottleneck	Not applicable	Increased under HO and THO relative to NO; highest under HO	HO vs. NO: ***; THO vs. NO: *; HO vs. THO: **	Not applicable; qRT-PCR-only validation	Included to compare HIF isoform responses; suggests possible HIF isoform divergence but requires HIF-2α protein validation

Abbreviations: DEG, differentially expressed gene; HO, sustained hypoxia; NO, normoxia; THO, transient hypoxia; RT-qPCR, quantitative reverse transcription PCR; FC, fold change; adj. P, adjusted P value; ns, not significant. Statistical notation: *P < 0.05, **P < 0.01, ****P < 0.001, ****P < 0.0001.

**FIGURE 6:**
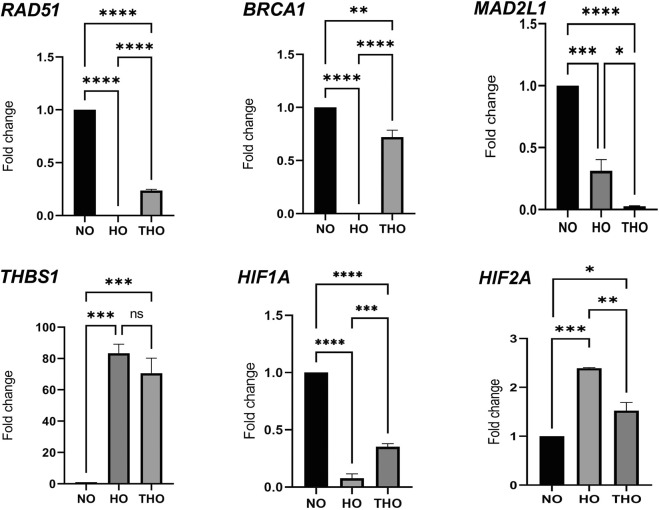
RT-qPCR validation of prioritised hub and bottleneck genes in gADSCs. Relative mRNA expression of selected hub and bottleneck genes was analysed in goat adipose tissue-derived mesenchymal stem cells (gADSCs) cultured under normoxia (NO), sustained hypoxia (HO), and transient hypoxia (THO). The validated genes included RAD51 recombinase (RAD51), BRCA1 DNA repair-associated (BRCA1), mitotic arrest deficient 2-like 1 (MAD2L1), thrombospondin 1 (THBS1), hypoxia-inducible factor 1-alpha (HIF1A) and hypoxia-inducible factor 2-alpha (HIF2A). Expression levels were normalised using housekeeping genes, ACTB and RPLP0, and relative expression was calculated using the 2^−ΔΔCt method. Data are presented as mean ± SEM from three independent biological replicates, with technical replicates for each reaction. Statistical significance is indicated as *P < 0.05, **P < 0.01, ****P < 0.001, and ****P < 0.0001; ns, not significant.

Footnote: RNA-seq, log_2_FC, values are shown where available in the main manuscript tables; complete RNA-seq, fold-change and adjusted P values for all validated genes are provided inSupplementary Material S2. RT-qPCR, fold-change direction and significance are based on Figures 5, 6. Because some RNA-seq, and RT-qPCR, comparisons differ in comparison orientation and because RT-qPCR, validates selected pairwise contrasts relative to NO, concordance was interpreted as concordant, partially concordant, discordant, or not validated.

### 3.7 RT-qPCR validation of hub and bottleneck genes

To further validate candidate regulatory nodes identified through protein-protein interaction (PPI) analysis, four overlapping hub/bottleneck genes were selected for RT-qPCR validation across normoxia (NO), sustained hypoxia (HO), and transient hypoxia (THO). In the THO vs. NO comparison, *RAD51* recombinase (*RAD51*), *BRCA1* DNA repair-associated (*BRCA1*), and mitotic arrest deficient 2-like 1 binding protein (*MAD2L1*) were identified as candidate hub/bottleneck-associated genes. In the THO vs. HO comparison, thrombospondin 1 (*THBS1*) was prioritised as a key hub/bottleneck gene associated with extracellular matrix and hypoxia-responsive signalling (Table 6, 7; Figure 6).

*RAD51* expression was significantly downregulated in gADSCs cultured under HO and THO compared with NO (P < 0.0001). Expression was significantly lower under HO than under THO (P < 0.0001), suggesting stronger suppression of homologous recombination-associated DNA repair signalling under sustained hypoxia (Figure 6). Similarly, *BRCA1* expression was significantly reduced under both HO and THO compared with NO (P < 0.0001), while its expression was significantly higher under THO than under HO (**P < 0.01), indicating partial recovery or differential regulation under transient hypoxia (Figure 6).

*MAD2L1* expression was significantly reduced under HO and THO compared with NO, with a greater reduction under THO. Expression was significantly lower in HO than NO (***P < 0.001), lower in THO than HO (P < 0.05), and markedly lower in THO than NO (***P < 0.0001) (Figure 6). These results suggest that genes associated with mitotic checkpoint control and chromosomal stability are differentially regulated by oxygen exposure in gADSCs.

In contrast to the DNA repair and checkpoint-associated genes, *THBS1* expression was markedly increased under both HO and THO compared with NO (***P < 0.001). No significant difference was observed between HO and THO (P > 0.05), indicating that *THBS1* induction may be a common response to low-oxygen culture rather than a feature specific to transient or sustained hypoxia (Figure 6). Collectively, these RT-qPCR validation data indicate that hub and bottleneck genes involved in DNA repair, mitotic regulation, extracellular matrix signalling, and hypoxia-associated adaptation are differentially regulated across oxygen conditions in gADSCs.

*HIF1A* expression was significantly reduced under HO and THO compared with NO (P < 0.0001), with significantly higher expression under THO than HO (P < 0.001). In contrast, *HIF2A* expression was significantly increased under HO compared with NO (P < 0.001) and THO (P < 0.01). Expression under THO was also significantly higher than under NO (P < 0.05), but lower than under HO, indicating that both sustained and transient hypoxia induced *HIF2A*, with sustained hypoxia producing a stronger response. This pattern suggests differential transcriptional regulation of hypoxia-inducible factor genes in gADSCs, with *HIF2A* showing hypoxia-associated induction and *HIF1A* showing reduced mRNA abundance under low-oxygen conditions.

Collectively, the RT-qPCR validation supports oxygen-dependent modulation of selected DEGs and indicates that sustained and transient hypoxia differentially regulate genes associated with pH regulation, stress response, metabolic adaptation, angiogenic signalling, and hypoxia-response pathways in gADSCs.

## 4 Discussion

This study examined transcriptional remodelling in goat adipose tissue-derived mesenchymal stem cells (gADSCs) cultured under normoxia (NO), transient hypoxia (THO), and sustained hypoxia (HO). The isolated cells showed expected MSC-like morphology, trilineage differentiation potential, high expression of MSC-associated surface markers, and low expression of hematopoietic/endothelial markers, supporting their suitability for transcriptomic analysis. RNA-seq and RT-qPCR analyses indicated that oxygen exposure is associated with changes in genes linked to cell-cycle regulation, DNA repair, extracellular matrix organisation, inflammatory signalling, pH regulation, angiogenic signalling, and hypoxia-inducible factor-associated adaptation. However, the three culture conditions are not equivalent manipulations of a single variable, and the conclusions must therefore be interpreted as condition-associated transcriptional responses rather than direct effects of oxygen tension alone.

### 4.1 Interpretative constraints arising from the oxygen-exposure design

The NO and THO groups were time-matched short-term exposures for 72 h at 21% O_2_ and 2% O_2_, respectively; therefore, this comparison most directly reflects the effect of oxygen concentration over the same duration. In contrast, HO cultures were maintained at 2% O_2_ for approximately three passages and therefore differed from THO not only in oxygen exposure but also in duration, passaging history, cumulative cell expansion, and potential adaptation to culture. In addition, HO cultures were briefly exposed to atmospheric oxygen during routine passaging for approximately 15–20 min before being returned to hypoxia. Repeated cycles of hypoxia, short reoxygenation, and re-exposure to hypoxia could alter cellular redox state, HIF dynamics, and stress-response gene expression. Thus, the HO vs. THO and HO vs. NO comparisons should be interpreted as comparisons of sustained versus intermittently passaged hypoxic adaptation rather than controlled single-variable oxygen comparisons.

This limitation is relevant because *in vitro* oxygen biology is strongly influenced by technical factors that are often underreported, including incubator/chamber set point, gas equilibration, medium depth, culture vessel geometry, cell density, oxygen consumption rate, and the difference between ambient gas-phase oxygen and the pericellular oxygen experienced by cells, as described (Place et al., 2017; Al-Ani et al., 2018; Pavlacky and Polak, 2020). Therefore, although the culture system was set to 2% O_2_ to create hypoxic conditions, dissolved/pericellular oxygen was not directly measured in the present study. This restricts the ability to conclude that all cells experienced identical oxygen tension throughout the experiment, particularly during HO passaging and re-equilibration.

### 4.2 Oxygen-dependent transcriptional remodelling in gADSCs

The transcriptomic data indicate that gADSCs do not respond to oxygen tension through a uniform hypoxia-associated programme. THO was associated with pathways related to DNA replication, cell-cycle regulation, base-excision repair, and sterol metabolism, whereas THO vs. HO showed enrichment of genes associated with cell motility, tissue remodelling, extracellular matrix organisation, wound healing, inflammatory response, and responses to oxygen-containing compounds. These findings are consistent with the view that short-term hypoxic exposure may induce an acute adaptive state, whereas sustained/intermittently passaged hypoxia may reflect longer-term remodelling and cellular acclimatisation. The absence of significant Gene Ontology enrichment among unique HO vs. NO DEGs should not be interpreted as the absence of a biological effect; rather, it may reflect the distribution of HO-responsive genes across multiple biological categories, the dataset’s statistical constraints, and incomplete functional annotation of *Capra hircus* genes and interactomes.

Under HO, genes such as eva-1 homolog C (*EVA1C*), mucin 13 (*MUC13*), ankyrin repeat and C-terminal domain containing protein (*ANKAR*), complement factor I (*CFI*), *HES6*, and *ERICH3* were identified. *EVA1C* has been associated with immune cell infiltration and neuronal survival in hypoxic-ischaemic models, suggesting a possible link to stress and immune response pathways, although its role in goat ADSCs remains unknown (Hu and Qu, 2021; Hu et al., 2022). *MUC13* has been implicated in hypoxia-associated metabolic adaptation (Kumari et al., 2018), *ANKAR* belongs to an ankyrin-repeat protein family involved in protein interactions and ubiquitylation-related processes (Kane and Spratt, 2021), *CFI* participates in complement regulation (Okroj et al., 2015; Verstockt et al., 2018), *HES6* has been linked to erythropoiesis, hypoxia/Notch signatures, and immune infiltration contexts (Wang et al., 2023; Irshad et al., 2015; Chen et al., 2024), and *ERICH3* has been associated with vesicular biology and treatment-response phenotypes (Liu et al., 2021).

Under NO, genes including CD96, *SIDT1*, *FGF12*, *SST*, *HOXD13*, *SAXO1*, *KCNT1*, and *NOXA1* showed condition-associated expression. These genes are linked to immune regulation, RNA transport, cellular release and survival pathways, developmental patterning, ciliary microtubule stability, ion-channel function, and reactive oxygen species generation (Georgiev et al., 2018; Shih and Hunter, 2011; Biadun et al., 2024b; Biadun et al., 2024a; Komitova et al., 2013; Kumar and Grant, 2010; Capecchi, 1996; Dacheux et al., 2015; Gertler et al., 1993; Møller et al., 2015; Honjo et al., 2008). Their association with NO may reflect the maintenance of baseline developmental, cytoskeletal, ion-channel, and redox-related functions under standard oxygen culture conditions.

THO-associated genes included *KNG1*, *LRP2*, *MERG*, *NFE2*, *FRZB*, *CSMD2*, *ECSCR*, and *AREG*. *KNG1* has been linked to thrombosis and renin-angiotensin-aldosterone system regulation (Zhang et al., 2019), *LRP2* encodes megalin, a receptor involved in ligand uptake and cellular development (Rasmussen et al., 2023), *MERG* is associated with phenylmercury resistance mechanisms (Masako and Hidemitsu, 1999), *FRZB* inhibits Wnt signalling and has been implicated in cartilage homeostasis (Leijten et al., 2012), CSMD-family proteins have been linked to diverse disease-associated signalling contexts (Ermis and Bell, 2022), *NFE2* is associated with erythroid regulation and HIF-related polycythaemic disorders (Kapralova et al., 2014), *ECSCR* modulates endothelial-cell functions and insulin sensitivity (Akakabe et al., 2013), and *AREG* has been connected with non-canonical HIF-2alpha-linked autocrine signalling (Stiehl et al., 2012).

In pairwise DEG interpretation, *PTGS1*, *CA12*, *ADCYAP1*, *RASGRF2*, *CABCOCO1*, MACROH2A2, *RAB31*, and *IFRD2* provide biologically relevant leads. *PTGS1* participates in prostaglandin biosynthesis, inflammatory signalling, vascular homeostasis, and repair-associated processes (Wang et al., 2019a). *CA12* contributes to pH regulation and extracellular matrix homeostasis and can be regulated by hypoxia in a context-dependent manner (Chen et al., 2016). *ADCYAP1*/PACAP has been linked to neuroprotection and anti-inflammatory activity (Rodriguez-Duboc et al., 2023; Nakamachi et al., 2016), *RASGRF2* is involved in Ras signalling and migration/invasion-related pathways (Lu et al., 2019), *CABCOCO1* has been associated with ciliary/flagellar biology (Kawashima et al., 2016), MACROH2A2 is involved in chromatin organisation and stemness-related epigenetic regulation (Nikolic et al., 2023), *RAB31* has been linked to hypoxia-induced apoptosis and survival pathways in adipose-derived stem cells (Ho et al., 2022), and *IFRD2* regulates lipid metabolism and Wnt signalling as a negative adipogenesis regulator (Vietor et al., 2023).

### 4.3 Validated DEGs and RNA-seq/qRT-PCR concordance

RT-qPCR validation confirmed oxygen-associated regulation of several candidate genes, including *CA12*, *CALR*, *DPP4*, *GADD45B*, *PARP*, *VEGFA*, *SNAI1*, *WIF1*, *HIF1A*, *HIF2A*, *RAD51*, *BRCA1*, *MAD2L1*, and *THBS1*. The validation results supported oxygen-associated changes for *DPP4*, *GADD45B*, *CALR*, *PARP*, *RAD51*, *BRCA1*, *MAD2L1*, *THBS1*, *HIF1A*, and *HIF2A*. However, not all RNA-seq-selected candidates were confirmed by RT-qPCR. The side-by-side RNA-seq and RT-qPCR comparison further indicated that not all RNA-seq-selected genes were validated concordantly by RT-qPCR (Table 8). In particular, *CA12* showed discordant directionality between RNA-seq and RT-qPCR, while *SNAI1* and *WIF1* were not significantly modulated by RT-qPCR. These differences may reflect platform-specific variation, primer- or isoform-specific effects, read-count modelling, donor-level variability, or sampling kinetics. Therefore, *CA12*, *SNAI1*, and *WIF1* should be interpreted with caution until confirmed using independent primer sets and protein-level or functional assays.

*DPP4* expression was significantly upregulated under NO and reduced under HO and THO, suggesting a potential shift in metabolic or immunomodulatory state during low-oxygen adaptation. *GADD45B* was reduced under hypoxic conditions, consistent with altered stress-response and cell-cycle-associated signalling. *CALR* and *PARP* were preferentially increased under THO, supporting the interpretation that transient hypoxia engages acute stress-buffering, endoplasmic-reticulum-associated, and DNA-stress-associated responses. These observations provide candidate markers for future mechanistic work but do not by themselves establish functional consequences.

The restored citation context supports interpretation of the RT-qPCR targets. *CA12* contributes to reversible CO2 hydration, bicarbonate generation, proton balance, and pH homeostasis (Türeci et al., 1998). *DPP4* is involved in glucose metabolism, insulin regulation, immune modulation, and the cleavage of X-proline dipeptides (Kameoka et al., 1993). *GADD45B* participates in stress signalling, DNA damage response, apoptosis, and cell-cycle control (Magimaidas et al., 2016). *CALR* may reflect endoplasmic-reticulum-associated protein folding, calcium homeostasis, and stress-buffering pathways, whereas *PARP* may indicate engagement of poly (ADP-ribosyl)ation, inflammatory signalling, and DNA-stress responses (Alemasova and Lavrik, 2019; Sawaya et al., 2023).

For the non-validated targets, *SNAI1* and *WIF1* should be interpreted cautiously. *SNAI1* belongs to the Snail family of developmental transcription factors (Murray et al., 2007), whereas *WIF1* encodes a secreted Wnt antagonist that binds Wnt proteins and inhibits Wnt signalling (Hsieh et al., 1999; Malinauskas et al., 2011). Their lack of significant RT-qPCR modulation in this study, therefore, indicates non-validation under the tested oxygen conditions, not absence of biological relevance.

### 4.4 *VEGFA* downregulation under THO

The significant reduction in *VEGFA* under THO is unexpected, as *VEGFA* is commonly induced by hypoxia through HIF-associated angiogenic signalling (Shweiki et al., 1992). Several biological explanations are possible, including time-dependent *VEGFA* mRNA kinetics, donor variability, goat ADSC-specific regulation, or transient uncoupling between HIF activity and *VEGFA* transcription. However, technical and cell-state explanations must also be considered. RNA degradation, suboptimal maintenance of low oxygen in the culture system, incomplete medium equilibration, reoxygenation during handling, or hypoxia-associated cell stress/death could contribute to lower *VEGFA* transcript abundance. In the present study, RNA quality was acceptable for sequencing, but no direct measurements of dissolved/pericellular oxygen, HIF-1α/HIF-2α protein stabilisation, cell death, or VEGF-A secretion were performed. Therefore, the *VEGFA* finding should be considered a transcript-level observation that requires confirmation by VEGF-A protein ELISA, endothelial migration/tube formation assays, and direct assessment of cell viability/stress under THO.

### 4.5 Hub and bottleneck genes

PPI analysis prioritised *RAD51* recombinase (*RAD51*), *BRCA1* DNA repair-associated (*BRCA1*), the mitotic arrest deficient 2-like 1 (*MAD2L1*), mitotic checkpoint-associated axis, thrombospondin 1 (*THBS1*), and hypoxia-inducible factor 1-alpha (*HIF1A*) as candidate regulatory nodes. *RAD51* and *BRCA1* are central components of homologous recombination-mediated DNA repair, and their reduced expression under HO and THO suggests that hypoxic culture may influence genome-maintenance pathways in gADSCs (Bindra et al., 2004; Bindra et al., 2005). The reduction in the mitotic checkpoint-associated transcript under hypoxia suggests altered proliferative or chromosomal-stability signalling (Wang et al., 2019b). *THBS1* was strongly increased under HO and THO, indicating that low oxygen may modulate extracellular matrix-associated signalling in gADSCs (Zhou et al., 2024). These network-based findings should be considered prioritisation results rather than direct evidence of functional control, particularly because goat protein-interaction databases are less complete than those for humans or mice.

### 4.6 *HIF1A*/*HIF2A* interpretation

The RT-qPCR data revealed divergent regulation of *HIF1A* and *HIF2A*. *HIF1A* mRNA was reduced under HO and THO, whereas *HIF2A* mRNA was increased under both hypoxic conditions, with the highest expression under HO. This result should not be interpreted as evidence of reduced HIF-1α signalling under hypoxia, because HIF-1α activity is regulated predominantly at the protein level through oxygen-dependent stabilisation, hydroxylation, degradation, and nuclear localisation (Semenza, 2000). HIF-1α and HIF-2α also have overlapping yet distinct regulatory roles, and prolonged hypoxia can promote a transition from HIF-1-dominant to HIF-2-dominant signalling in some systems (Keith et al., 2011; Jaskiewicz et al., 2022). Thus, the reduced *HIF1A* transcript level and increased *HIF2A* transcript level in gADSCs suggest possible HIF isoform divergence during sustained hypoxic adaptation, but this interpretation requires confirmation by HIF-1α and HIF-2α protein quantification, nuclear localisation, and downstream target activation assays.

### 4.7 Limitations and future directions

This study has several limitations. First, the RNA-seq experiment included three biological donors, which is appropriate for exploratory transcriptomic profiling but limits power to fully characterise donor-specific variation. Second, the use of two-dimensional monolayer culture and 10% FBS may influence ADSC gene expression and may not fully reproduce the native adipose tissue niche or xeno-free therapeutic culture conditions. Third, the experimental design included time-matched NO and THO groups but not a fully time- and passage-matched HO comparator. HO cultures were exposed to 2% O_2_ for several passages and briefly to atmospheric oxygen during passaging, whereas THO cultures were exposed to 2% O_2_ for 72 h. Therefore, HO comparisons include effects of oxygen tension, exposure duration, passaging, culture adaptation, and intermittent reoxygenation.

Fourth, the achieved and maintained oxygen concentration at the cellular level was not directly measured. Future studies should use oxygen sensors or dissolved/pericellular oxygen monitoring to confirm chamber performance, medium equilibration, and oxygen stability during handling. Fifth, although *ACTB* and *RPLP0* were used together for RT-qPCR normalisation, a formal stability analysis of multiple candidate reference genes across NO, HO, and THO was not performed. Future work should evaluate reference genes using algorithms such as geNorm, NormFinder, or BestKeeper and report stability metrics. Sixth, RNA-seq/qRT-PCR discordance for some genes, including *CA12*, and non-significant validation for *SNAI1* and *WIF1*, indicate that transcript-level findings should be interpreted with caution. Additional validation using independent primer sets, protein assays, and functional readouts is needed.

Future experiments should include time- and passage-matched designs, such as NO-72 h vs. THO-72 h and NO-three passages vs. HO-three passages, with minimal or controlled reoxygenation during passaging. Additional assays should assess proliferation, apoptosis, senescence, reactive oxygen species generation, mitochondrial function, DNA damage response, homologous recombination activity, *VEGFA* secretion, endothelial tube formation, and HIF-1α/HIF-2α protein stabilisation and nuclear localisation. These studies would determine whether the transcriptomic changes identified here translate into functional alterations relevant to ADSC biology and regenerative applications.

## 5 Conclusion

In conclusion, this study provides a transcriptomic resource describing how gADSCs respond to three distinct oxygen-associated culture conditions: short-term normoxia, short-term transient hypoxia, and sustained/intermittently passaged hypoxia. The NO vs. THO comparison most directly reflects a 72 h oxygen-concentration contrast, whereas comparisons involving HO reflect combined effects of low oxygen, longer exposure duration, repeated passaging, culture adaptation, and brief reoxygenation during handling. Therefore, the data should be interpreted as condition-associated transcriptional remodelling rather than as evidence of oxygen tension as the sole causal variable.

The study identifies candidate genes and pathways associated with DNA repair, cell-cycle regulation, extracellular matrix signalling, stress adaptation, pH regulation, angiogenic signalling, and HIF-associated responses. RT-qPCR validation supports oxygen-associated regulation of several targets, including *DPP4*, *GADD45B*, *CALR*, *PARP*, *VEGFA*, *HIF1A*, *HIF2A*, *RAD51*, *BRCA1*, *MAD2L1*, and *THBS1*, while also revealing non-validation or discordance for some RNA-seq-selected genes. The divergent *HIF1A* and *HIF2A* mRNA patterns suggest possible HIF isoform-specific adaptation, but HIF signalling must be confirmed at the protein and functional levels. Overall, these findings provide hypotheses for future mechanistic studies, but definitive biological and translational conclusions will require controlled oxygen monitoring, time- and passage-matched experimental designs, protein-level validation, and functional assays.

## Data Availability

The datasets presented in this study can be found in online repositories. The names of the repository/repositories and accession number(s) can be found in the article/Supplementary Material.
