# Supplementary material for: Transcriptome analysis of goat adipose tissue-derived mesenchymal stem cells cultured in variable oxygen conditions
Source: Front Cell Dev Biol. 2026 Jul 1;14:1814793. doi: 10.3389/fcell.2026.1814793 (PMC13368766; doi:10.3389/fcell.2026.1814793)
Supplement: Supplementary file 1 [file DataSheet3.docx]

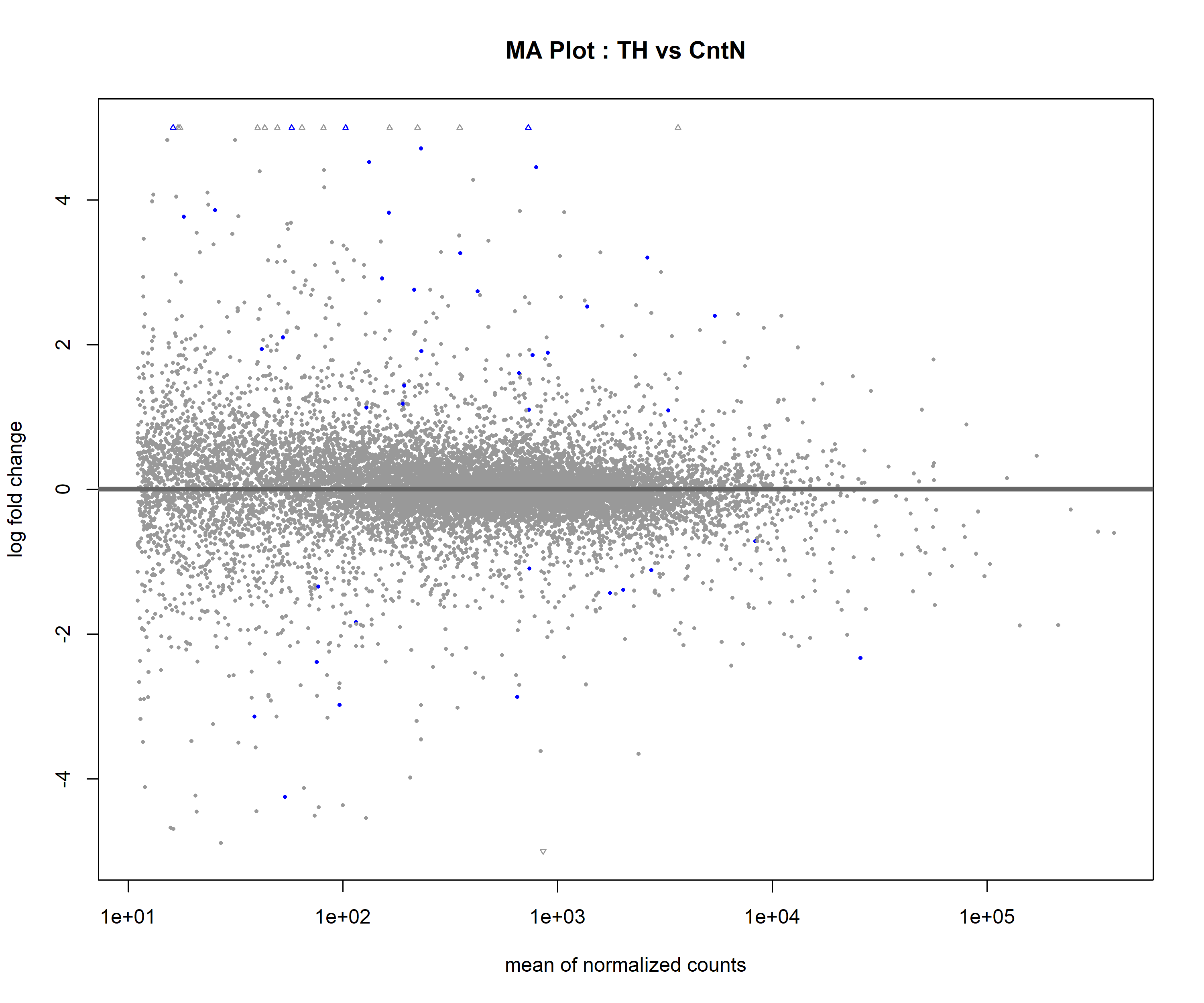

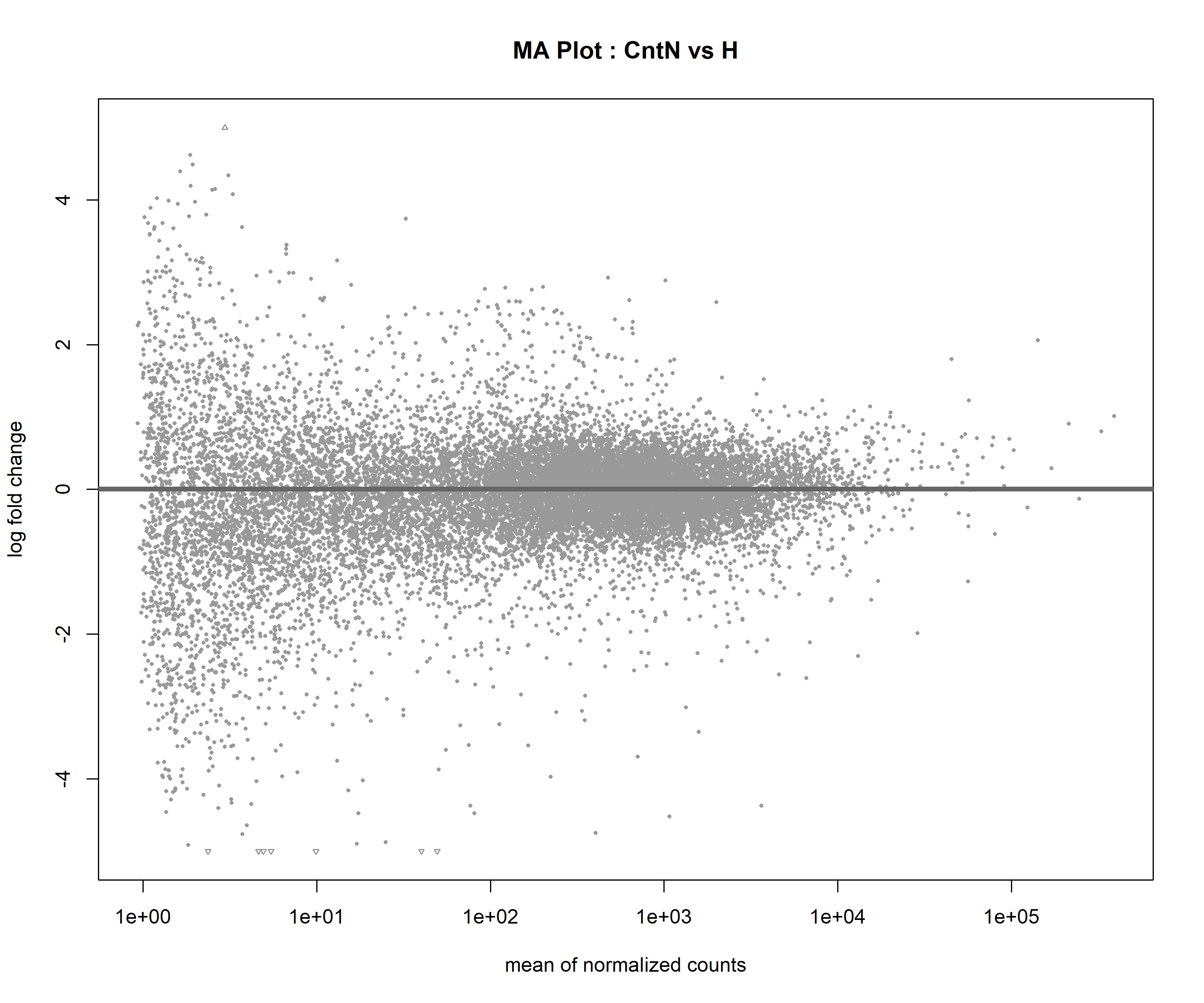


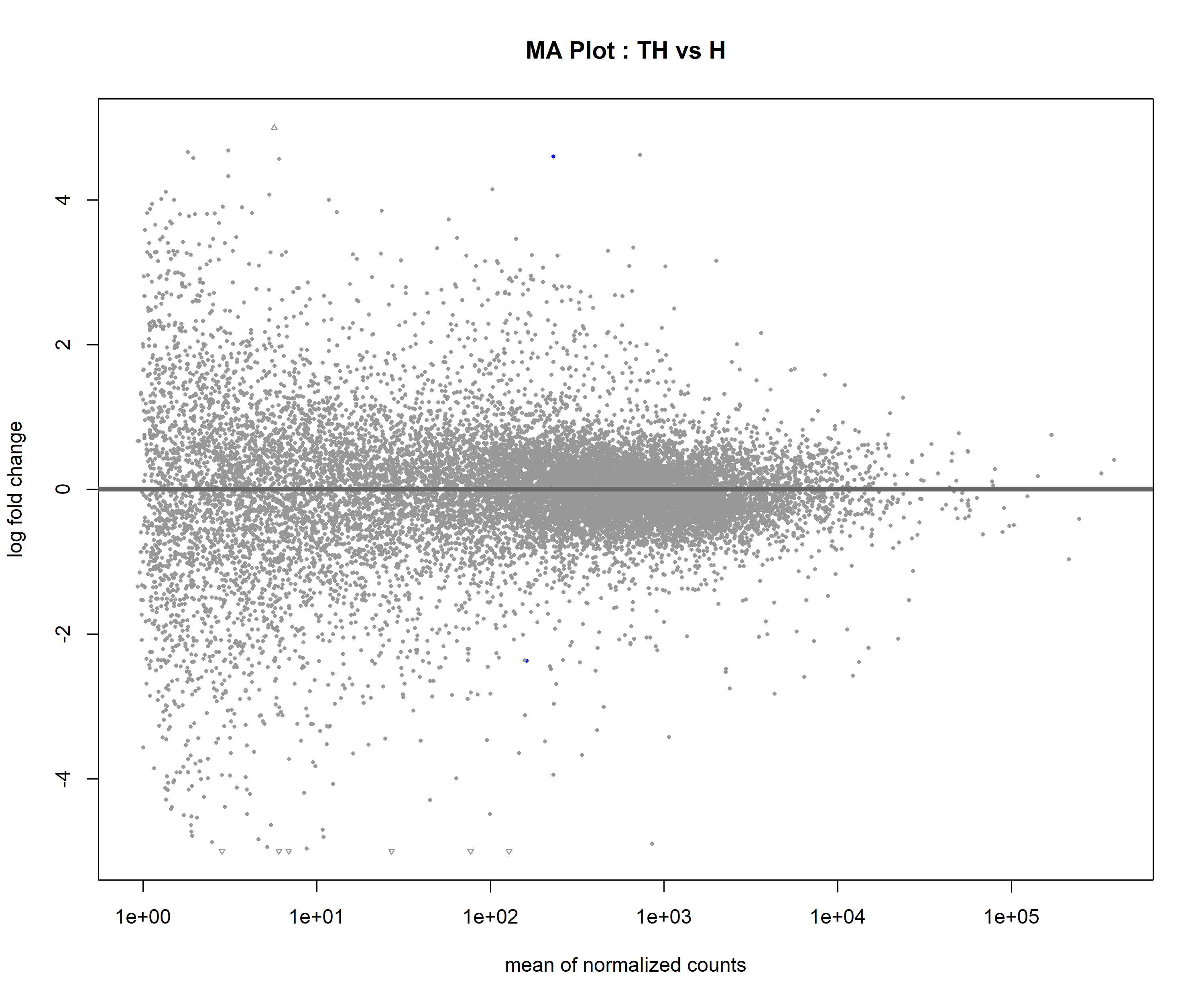


Figure S1A. MA plots showing differential gene expression across oxygen-culture comparisons in gADSCs.


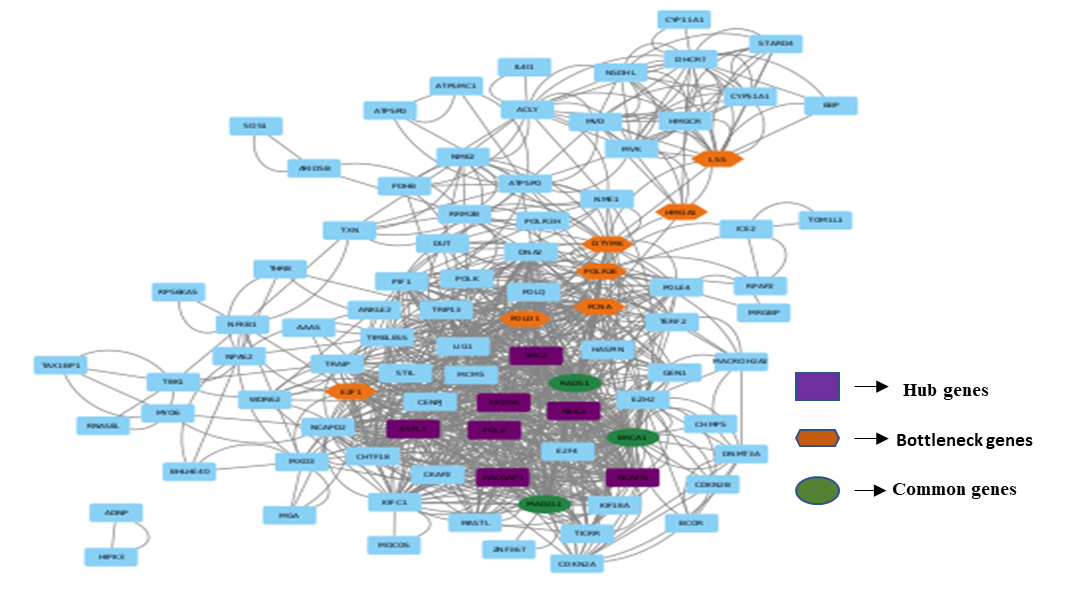


Figure S1B. The protein-protein interaction network shows the hub (purple), bottleneck (orange), and genes with both features (green) for THO vs. NO.


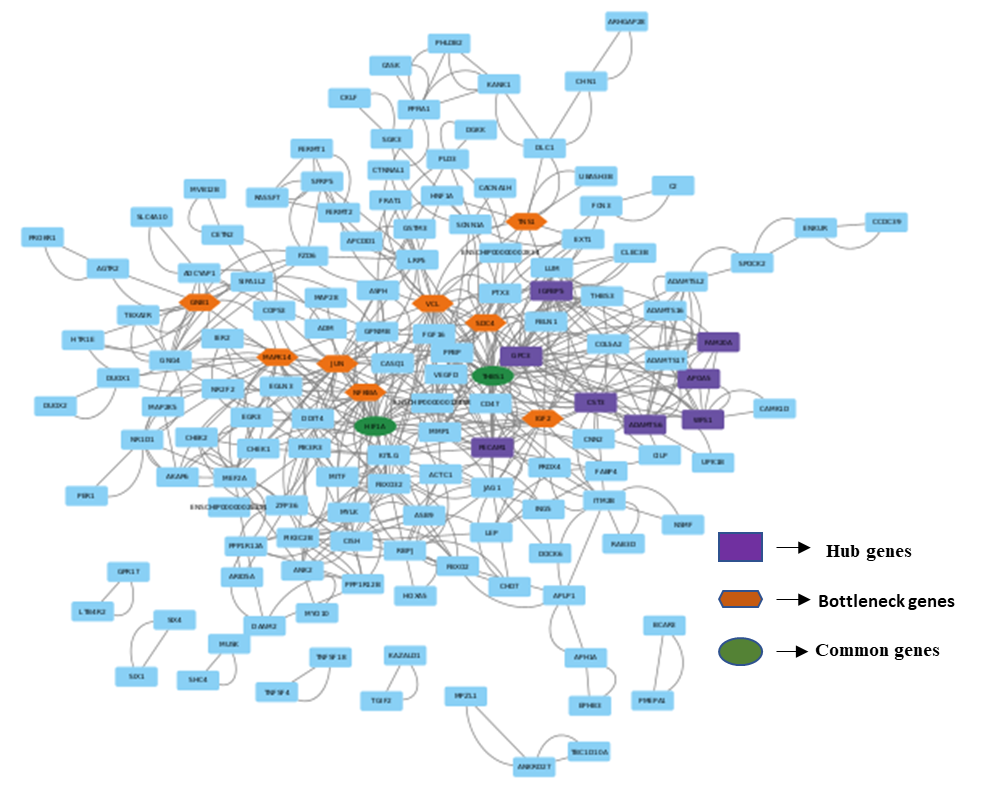


Figure S1C. The protein-protein interaction network shows the hub (purple), bottleneck (orange), and genes with both features (green) for THO vs. HO treatment.
